# A governance process lens for evaluating local public sport facilities: a multi-level conceptual framework

**DOI:** 10.3389/fspor.2026.1844897

**Published:** 2026-06-02

**Authors:** Xuanye Pan, Nor Eeza Zainal Abidin, Mohd Salleh Aman

**Affiliations:** Faculty of Sports and Exercise Science, University of Malaya, Kuala Lumpur, Malaysia

**Keywords:** conceptual framework, governance process, local public sport facilities, multi-level evaluation, organizational mediation, public steering

## Abstract

Local public sport facilities are not simply sites of infrastructure provision and service use. They are also shaped by governance through public steering, organizational mediation, and local decisions about resource allocation. Existing evaluation approaches remain fragmented. Studies often examine user experience, organizational performance, accessibility, or efficiency in relative isolation, with limited attention to how these concerns relate within local facility development. This study develops a multi-level conceptual framework for evaluating local public sport facilities through a governance process lens. The framework was derived through a structured conceptual synthesis of recurring strands in the literature and distinguishes three analytically distinct but operationally interdependent levels: community use, lived service experience, and user-valued outcomes at the micro level; organizational and managerial governance mechanisms at the meso level; and policy steering, resource allocation, accessibility, and system-level efficiency conditions at the macro level. Rather than proposing a universal scoring model, the framework provides an integrative architecture for understanding how these levels are related within the same public development process. It offers a governance-sensitive heuristic for future research, evaluation design, and local public steering.

## 1 Introduction

### 1.1 The problem of fragmented evaluation

Local public sport facilities are commonly discussed as sites of infrastructure provision, service delivery, and community participation. In policy and practice, attention often centers on construction, maintenance, utilization, access, and public investment. Yet these facilities are not simply physical assets or neutral venues in which sport and recreation take place. They are also shaped by governance processes through which public authorities define priorities, allocate resources, structure delivery arrangements, and determine how facilities should respond to community needs. In this sense, local public sport facilities are service sites embedded in wider systems of governance, public steering, and accountability.

Local public sport facilities are increasingly tied to broader public goals. They are expected to support physical activity while contributing to inclusion, community life, equitable access, and the effective use of public resources, reflecting wider concern with public value (1) and citizen-centered service assessment (2). As a result, local authorities and facility providers face growing pressure to show that facilities are available, relevant, publicly defensible, and responsive to community needs. Research on local sport policymaking points in the same direction by showing that policy development depends on local work processes, strategic interpretation, and the practical role of sport managers (3, 4).

Despite this, existing approaches to evaluating local public sport facilities remain fragmented. One line of research focuses on community use, lived service experience, and user-valued outcomes. A second examines facilities through organizational and managerial lenses, highlighting delivery arrangements, management capacity, and performance measurement. A third concentrates on efficiency and resource allocation, asking how public inputs are translated into outputs across facilities, municipalities, or systems. These traditions offer useful insights, yet they have largely developed in parallel without a shared conceptual structure.

This fragmentation matters because local public sport facilities cannot be reduced to a single evaluative object viewed from different angles. Across this literature, local public sport facilities have been examined in terms of spatial equity and accessibility (5), organizational structure and public-private relationships (6), perceived quality and user experience in public aquatic centers (7, 8), performance measurement and managerial perspectives in public leisure facilities (9, 10), and perceived service quality in public sports center contexts (11). In light of these, studies point to multiple ways of conceptualizing what is being evaluated. These are related but non-identical ways of defining the evaluative object.

This pattern reflects broader developments in sport and public sector research. Studies increasingly question unidimensional models and the limits of fragmented literatures, while also recognizing the difficulty of evaluating hybrid public organizations shaped by multiple criteria (12–14). Related work in sport policy likewise emphasizes linked macro-, meso-, and micro-level processes while keeping community central within wider political and organizational systems (15, 16). What remains underdeveloped, however, is a framework that connects these strands specifically for local public sport facilities.

Against this background, this study develops a multi-level conceptual framework for evaluating local public sport facilities through a governance process lens. The contribution is not to replace user-oriented, organizational, or efficiency-related approaches with a single dominant criterion. Rather, it is to clarify how these concerns define different but related levels of the same public development process. In this way, the article reconceptualizes the evaluative object and proposes an integrative architecture for future research and governance-sensitive evaluation.

### 1.2 How the conceptual framework was derived

This article takes the form of a conceptual analysis; it is not intended as a systematic or exhaustive review. The framework was developed through a structured conceptual synthesis of recurring strands in the literature on local public sport facilities and related work in public service evaluation, sport policy, and governance (14–16). The aim was to clarify analytically how different approaches define the evaluative object and what each approach makes visible, leaves underdeveloped, or treats as secondary.

More specifically, the framework was derived by comparing three broad and recurring lines of inquiry. The first approaches local public sport facilities through community use, lived service experience, and user-valued outcomes (7, 8, 11, 17). The second examines them through organizational and managerial arrangements, focusing on coordination, delivery structures, and governance mechanisms (6, 18–20). The third approaches them through policy steering, resource allocation, accessibility, and system-level efficiency conditions (5, 21–24). These strands were selected because they recur across the literature and collectively capture the main ways in which local public sport facilities have been conceptualized and assessed.

The framework emerged through an iterative comparison of these strands in terms of their underlying assumptions, principal evaluative focus, and limitations when used in isolation. This comparison showed that the field's main weakness lies not simply in the use of different indicators, but in the absence of a shared evaluative logic capable of relating user-valued outcomes, organizational mediation, and wider policy-resource and public-purpose conditions within the same conceptual structure (12, 14).

The proposed framework was developed in response to that gap. It does not aggregate existing concerns into a single flat model, but organizes them across analytically distinct and operationally interdependent levels. In this sense, the framework should be understood as a governance-oriented conceptual architecture developed through structured synthesis and conceptual mapping across fragmented traditions.

## 2 Fragmented traditions in evaluating local public sport facilities

### 2.1 Community use, lived experience, and user-valued outcomes

One important line of research evaluates local public sport facilities through community use and user-valued outcomes. This orientation fits broader developments in public service research, where greater attention has been given to citizen-centered evaluation, value creation, and the role of user experience in assessing publicly governed services (1, 2). Within this line of work, facilities are assessed according to how they are encountered in practice, including how users perceive service quality (11), accessibility (25) and satisfaction (26). This shift matters because it moves attention away from provision counts and provider-defined indicators toward the lived realities of public service use. It also makes clear that local public sport facilities are not just built environments or administrative units. They are public service settings in which value is experienced, interpreted, and judged in everyday interaction.

This perspective is particularly relevant in local public service contexts. The existence of infrastructure does not guarantee that facilities are experienced as meaningful, welcoming, or responsive. User-centered evaluation needs to be situated within wider public value commitments, since it cannot be reduced to a purely consumer-oriented logic (27). A facility may be well resourced on paper yet still be perceived as inconvenient, poorly maintained, socially uninviting, or misaligned with local activity needs. In this sense, user-centered evaluation plays a corrective role by reminding scholars and practitioners that facilities derive practical legitimacy from being valued in use, not merely from being provided. Within sport management, this emphasis on lived evaluation is compatible with experience quality research, which treats users’ assessments of service environments as multidimensional and not reducible to a single satisfaction score (17).

This line of work is, nevertheless, incomplete on its own. User-valued outcomes show how facilities are experienced, but they do not fully explain how those experiences are produced, how they are sustained organizationally, or how they are shaped by broader governance conditions. Public service research makes a similar point. Zhang et al. (28) show that citizen satisfaction is shaped not only by perceived performance, but also by expectations, disconfirmation, and contextual variation. For this reason, satisfaction cannot function as a self-sufficient proxy for overall facility performance. Community use, lived experience, and user-valued outcomes provide indispensable evidence. They capture only one part of the evaluative problem and leave the conditions that produce these experiences underexplained.

### 2.2 Organizational and managerial approaches to facility evaluation

A second tradition approaches local public sport facilities through organizational and managerial lenses. Typically, the main concern is how facilities are coordinated (29), managed (30), and assessed (19) as public service arrangements, rather than how they are experienced by users. This tradition emphasizes delivery structures, managerial capacity, internal coordination, operational routines, and the institutional mechanisms through which facilities are maintained and adapted over time.

In practice, this translation occurs through concrete organizational processes such as staffing, maintenance, scheduling, budgeting, and the coordination of roles among local authorities, facility managers, and community sport organizations, all of which shape how facilities operate and how services are encountered by users (31). From this perspective, organizational and managerial evaluation captures a distinct analytical level by examining the performance arrangements and accountability structures through which local public sport facilities are run. This level keeps attention on organizational mediation, without reducing performance to user satisfaction or system-level efficiency alone (1, 18).

The relevance of this perspective is reinforced by the public sector performance literature. De Waele et al. (13) argue that hybrid public sector organizations operate under multiple and sometimes conflicting institutional logics, making performance difficult to capture through one dominant criterion. They propose that performance frameworks must account for possible synergies and frictions among dimensions such as economy, efficiency, effectiveness, and equity. This insight is highly relevant to local public sport facilities, which are often expected to be community-oriented, efficient, publicly accountable, and organizationally viable. Similarly, van der Kolk (20) shows that what matters in performance measurement is not merely the presence of performance systems, but how such systems are designed, interpreted, and used. Applied to local facilities, this means that managerial and organizational evaluation should not be reduced to internal reporting or operational smoothness alone. It should be understood as the level at which governance is translated into organizational practice.

However, taken alone, this tradition also has limits. A strong focus on managerial arrangements may become too organization-centered, giving insufficient attention to whether facilities are experienced positively in community life or whether they are aligned with broader local priorities. Organizational performance is therefore necessary to understand, but not sufficient to define local public sport facility development in full.

### 2.3 Resource allocation and efficiency-oriented evaluation

A third tradition approaches local public sport facilities through resource allocation and efficiency. Here facilities are treated as public investments whose use of scarce resources must be justified through outputs and outcomes that can be compared across facilities, municipalities, or systems. Sport facilities research already illustrates this system-level concern, whether through studies of the operational efficiency of English public sport facilities (23), policy-oriented work on facility utilization strategies (32), or recent analyses of national fitness public service efficiency in China (21).

This tradition adds a system-level perspective that neither user-centered nor organization-centered approaches can provide on their own. It asks whether facilities are funded and distributed in defensible ways, whether resource conversion differs across jurisdictions, and whether allocation patterns align with public priorities. It also connects efficiency to broader questions of equity and service coverage in public sport facilities rather than to technical performance alone.

Yet this perspective also involves simplification. When evaluation privileges what is easiest to count, important experiential and organizational dimensions can recede from view. Bayle and Clausen (12), in a different sport governance context, argue that multidimensional and process-oriented models are needed when performance is shaped by multiple expectations, legitimacy demands, and interdependencies across inputs, throughput, outcomes, and feedback. A similar pattern can be observed in this context. System-level judgments become thin when resource conversion is isolated from the governance and service processes through which public value is produced.

### 2.4 Fragmentation and the governance gap

These traditions differ not only in their indicators or topical emphases, but in how they define the object of evaluation. They rest on different assumptions about what local public sport facilities are and how they should be evaluated. Some studies treat facilities primarily as lived public service environments (7, 8, 11), others as organizational arrangements to be managed and coordinated (6, 18, 19), and others as components of wider policy, allocation, and accessibility systems (5, 22, 24). These are not trivial shifts in emphasis. They indicate that the literature often evaluates different objects under the same general label of facility evaluation.

The consequence is fragmentation in both measures and the definition of what is being evaluated. Local public sport facilities are encountered in everyday use, while user experiences are mediated through organizations and shaped by wider policy-resource conditions. Organizational arrangements, in turn, are embedded in community use and public steering. Allocation decisions become meaningful when they support facilities that are usable, publicly valued, and aligned with community needs (5, 14, 33). In other words, these relationships suggest that local public sport facility development is better understood through a governance process lens than through any single evaluative tradition in isolation.

This move from fragmented evaluation to governance-sensitive conceptualization is consistent with broader developments in sport policy and community sport research. Rowe et al. (14) show that fragmented literatures can constrain understanding when practically related domains are examined separately. Related work similarly calls for sport-related interventions to be analyzed across interconnected processes (15), while retaining a clear focus on community within broader social, political, and organizational conditions (16). The central issue is not the need for more indicators, but the lack of a coherent evaluative architecture capable of relating distinct levels of local facility development.

## 3 From service sites to governance processes

### 3.1 Why local facility development should be understood as a governance process

The fragmented traditions reviewed above suggest that evaluating local public sport facilities is both a conceptual and methodological problem. Facilities are still often treated as self-contained service sites whose performance can be inferred from isolated indicators such as user satisfaction, managerial quality, or technical efficiency. This view understates the extent to which facilities are constituted through governance processes. They do not emerge, operate, or endure independently of public steering, institutional arrangements, resource decisions, and organizational mediation. Evaluation that begins only at the point of service encounter captures outcomes after governance has already structured much of what becomes possible.

A governance-process perspective shifts attention from isolated performance outcomes to the processes through which those outcomes are made possible. Local sport policy research points in the same direction: policy development depends on how local actors interpret priorities, organize processes, and translate broad ambitions into concrete practice (3), while turning points and path dependencies shape what becomes feasible over time (34). Local public sport facilities are better understood as governance arrangements, not simply as neutral infrastructure stocks or service delivery units.

This reconceptualization is consistent with wider work on hybrid sport and public organizations. Such facilities are expected to be accessible, community-oriented, efficient, publicly accountable, and organizationally viable. Under these conditions, evaluation cannot be reduced to one dominant dimension without distorting the object being assessed (12, 13).

This perspective also explains why local public sport facilities are tied to broader policy ambitions. Once facilities are justified through participation, inclusion, community benefit, and social value, evaluation cannot stop at whether they exist or how frequently they are used. It must also consider how those goals are interpreted and mediated through local governance arrangements (16).

The move from service sites to governance processes is more than rhetorical. It redefines the evaluative object. Facilities are no longer viewed simply as places where services happen; they are outcomes of public steering, stakeholder negotiation, organizational mediation, and resource allocation. Accordingly, meaningful evaluation has to begin before observable service outcomes and examine how those outcomes are enabled, constrained, or unevenly shaped across local contexts.

### 3.2 Public steering, organizational mediation, and community use

If local public sport facility development is understood as a governance process, three mediating domains come into view: public steering, organizational mediation, and community use. These are not interchangeable labels but analytically distinct components of the same evaluative chain. Public steering concerns the policy environment in which local authorities define priorities, allocate funds, set institutional expectations, and shape development pathways. Local public sport facilities are not governed against a neutral background. They are shaped by formal and informal steering devices, budgetary priorities, and local interpretations of what facilities are for and whom they should serve.

The second concerns organizational mediation. Even where local priorities are clear, they do not automatically translate into practice. They must be enacted through organizations, routines, managerial choices, and delivery structures. This is also evident in recent work on sport policy implementation, which shows that community sport organizations operate under strong external pressures and competing demands, while municipalities and other institutional actors shape organizational practice and facility access in consequential ways (35). This is why the organizational level cannot be collapsed into either policy intent or user response. In local public sport facilities, organizational and managerial arrangements mediate among administrative accountability, public accessibility, community responsiveness, and resource constraints. Evaluation needs to attend to how facilities are scheduled, staffed, coordinated, maintained, and adapted over time. Without this organizational lens, the governance process remains insufficiently explained.

The third concerns community use. Even when facilities are strongly shaped by policy and organization, they still acquire practical meaning in use. Community members do not encounter “policy” or “organizational design” in the abstract; they encounter facilities as places that either enable or constrain access, participation, social interaction, and everyday relevance. A governance-process account of local public sport facilities should not displace users in favor of structure. Conversely, it should explain how public steering and organizational mediation shape the conditions under which community use becomes possible, valued, or contested.

These three components are relationally connected, not arranged as a simple sequence. Public steering shapes organizational possibilities; organizational mediation influences the conditions under which facilities are encountered in practice; and community use feeds back into governance through claims about relevance, legitimacy, and need. The purpose is to preserve the distinction among these domains while showing that no single domain can represent the evaluative object as a whole. Local public sport facility evaluation accordingly requires a relational understanding of development.

### 3.3 The limits of single-dimensional evaluation

Single-dimensional evaluation persists in part because facility assessment is often organized around available metrics before the facility has been clearly conceptualized as a public object. Once evaluation begins from utilization rates, satisfaction scores, or efficiency ratios, facilities can easily appear as self-evident units of assessment. A governance-process view directly challenges that assumption.

Seen in this light, the limits of single-dimensional evaluation become clearer. Satisfaction-centered approaches capture lived experience, management-centered approaches illuminate organizational capacity, and efficiency-centered approaches benchmark resource conversion. These approaches are not incorrect; the problem is that none defines the evaluative object in full.

Evaluation choices also have political consequences. Assessing facilities solely through utilization, efficiency, or satisfaction privileges one interpretation of what facilities are for and what forms of success are legitimate. Hence, evaluation is part of governance, shaping which development pathways appear publicly defensible (15, 36).

Local public sport facilities are not best evaluated as isolated service sites, as their development is embedded in governance processes where public steering, organizational mediation, and community use are interwoven. This reconceptualization retains existing evaluative traditions while repositioning them within a wider structure: user-valued outcomes capture one part of the problem, organizational and managerial mechanisms another, and policy-resource conditions a third. The next section develops that architecture.

## 4 A multi-level conceptual framework for evaluating local public sport facilities

### 4.1 Conceptual rationale

The weakness of existing approaches lies less in the number of indicators they use than in the way they define what is being evaluated. Local public sport facilities are treated in the literature as service environments, organizational arrangements, investment cases, or components of wider public service systems, and each perspective illuminates a different aspect of development (5–10). The difficulty is that these perspectives are often treated as if they referred to the same analytical plane, or as if their accumulation alone could produce conceptual integration.

This article addresses that problem by organizing evaluation across analytically distinct levels. The aim is to clarify that the evaluative object is constituted through governance processes, not merely to add more dimensions. For that reason, the framework is best understood as a governance-process architecture rather than as a multidimensional indicator model.

A model can remain conceptually flat even when it appears multidimensional, especially when user experience, managerial quality, accessibility, and efficiency are grouped within one undifferentiated performance space. The present framework takes a different approach. It treats these concerns as addressing different but related levels of local facility development and therefore as answering different evaluative questions. In this respect, the framework repositions existing traditions within a clearer integrative structure (12, 14).

**Figure 1:**
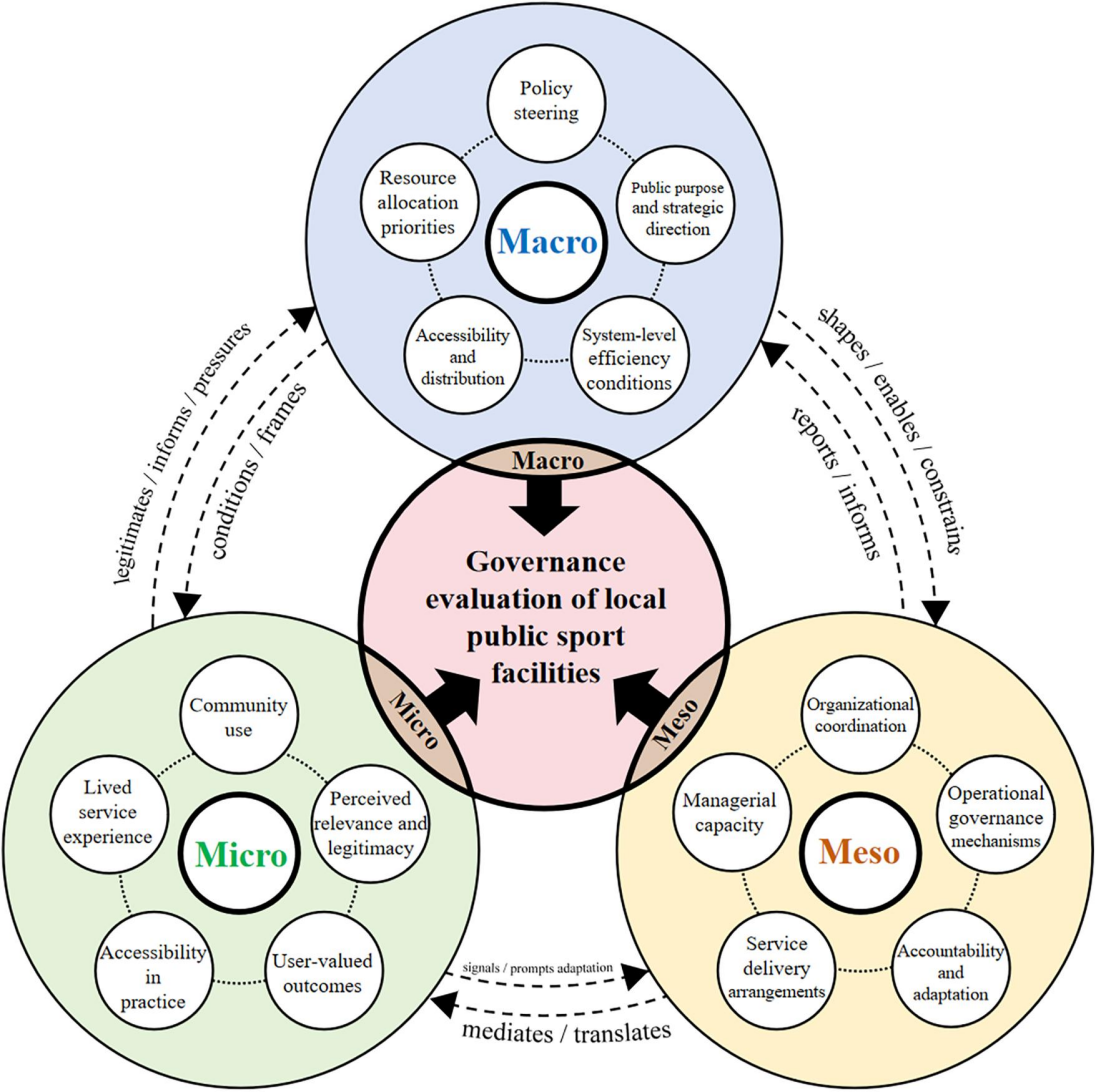
A governance process framework for evaluating local public sport facilities. The figure presents local public sport facility development as a governance process structured across three analytically distinct but operationally interdependent levels: macro-level policy steering and resource conditions, meso-level organizational and managerial governance mechanisms, and micro-level community use and user-valued outcomes. The arrows indicate that these relationships are not purely top-down. Macro-level conditions shape and frame organizational and user-level possibilities, meso-level mechanisms mediate how public priorities are translated into operational practice, and micro-level experiences and outcomes may feed back into organizational adaptation, public legitimacy, and policy learning.

It also builds on multi-level thinking in sport policy and community sport research, where macro-level policy design, meso-level implementation, and micro-level consequences are examined within broader political and organizational contexts (15, 16). Accordingly, the framework distinguishes three analytically distinct but operationally interdependent levels: community use and user-valued outcomes; organizational and managerial governance mechanisms; and policy steering, resource allocation, accessibility, and system-level efficiency conditions. Figure 1 visualizes this arrangement as a governance process architecture, showing how the three levels remain differentiated while linked through directional and recursive relationships.

### 4.2 Micro level: community use, lived service experience, and user-valued outcomes

The micro level concerns how local public sport facilities are encountered, interpreted, and evaluated in use. At this level, the facility appears primarily as a lived public service environment, while its role as a policy instrument or organizational arrangement remains in the background. The central evaluative concern is whether facilities generate experiences that are meaningful, accessible, relevant, and positively valued by users. Key aspects include lived service experience, perceived relevance, satisfaction, and the social-relational dimensions of community use.

The micro level matters because local public sport facilities acquire practical legitimacy through everyday community use. A facility may be well funded, strategically justified, and formally well governed, yet still fail to be experienced as useful, welcoming, or responsive. User-valued outcomes are not superficial signals; they show whether facilities generate value in practice beyond administrative design. Even so, this level is not self-sufficient. It reveals how facilities are experienced, while leaving open how those experiences are produced, sustained, or differentiated across governance conditions. At the micro level, the key evaluative question is how local public sport facilities are experienced and judged in everyday community use.

### 4.3 Meso level: organizational and managerial governance mechanisms

The meso level concerns the organizational and managerial governance mechanisms through which local public sport facilities are operated, coordinated, and publicly steered. It captures the level at which public expectations, local priorities, and resource constraints are translated into concrete forms of delivery. From this perspective, evaluation focuses on the quality and configuration of governance mechanisms, including managerial capacity, institutional coordination, delivery routines, accountability structures, staffing arrangements, and the processes through which facilities are maintained and adapted over time. Measurement is itself part of this governance work, insofar as facility utilization data may be used differently for learning, steering, and accountability purposes within managerial settings (37). This managerial dimension becomes especially visible under fiscal constraints, where the management of sport facilities is closely tied to prioritization, adaptation, and the practical consequences of limited resources (38).

This level is essential because public steering and community use cannot be adequately understood without examining how they are mediated through organizations. Public priorities do not directly become lived experiences; they are filtered through organizational actors, capacities, and routines. Recent research on sport facility managers further shows that organizational responses are shaped by tensions between historically dominant user groups and a growing policy focus on individual users, underscoring the importance of managerial mediation in facility governance (39). The meso level occupies an intermediate yet substantial position in the framework because it explains how governance becomes operational.

This organizational emphasis is strongly supported by work on hybridity and multidimensional performance measurement in public sector settings. De Waele et al. (13) argue that organizations operating under multiple administrative models and competing institutional demands require performance frameworks that can account for possible synergies and frictions among dimensions. Van der Kolk (20) further shows that public sector performance measurement depends on what is measured, how measurement systems are designed, and how they are used. Applied to local public sport facilities, this suggests that organizational and managerial governance mechanisms should be conceptualized not as a residual category, but as the level at which governance priorities are translated into practical conditions of delivery. The meso level brings a different question into focus: through what organizational and managerial mechanisms are user-valued outcomes shaped, delivered, and sustained?

### 4.4 Macro level: local policy steering, resource allocation, and system-level efficiency conditions

The macro level concerns the broader local policy and resource environment within which public sport facilities are developed, prioritized, and sustained. At this level, facilities are viewed primarily as objects of public steering embedded in wider allocation and accountability systems. The evaluative focus includes policy direction, allocation priorities, stakeholder mandates, strategic justifications, and system-level efficiency conditions across facilities, municipalities, or jurisdictions.

This level is necessary because local public sport facility development is embedded in policy processes that involve political choices, institutional priorities, and public justification. Ehnold et al. (15) show how public programs define problems, assign mandates, and reshape organizational machinery. In local facility contexts, this broader insight is reflected in rules, budget decisions, and public rationales about what facilities are for and whom they should serve. Sport facility research also shows that allocation choices are closely connected to questions of utilization, coverage, and equity (21, 32).

The macro level extends beyond policy aspiration by incorporating system-level efficiency conditions. These conditions should not be equated with technical efficiency analysis alone. They refer more broadly to how local governments allocate scarce resources, prioritize facilities, and assess whether public inputs are converted into publicly defensible outputs. Studies of public sport facilities and related national fitness services illustrate the value of this lens for examining resource conversion, utilization patterns, and broader allocation efficiency in sport-specific settings (21, 23). At the macro level, the central evaluative question is how local public sport facilities are shaped by policy steering, allocation priorities, and system-level efficiency conditions within the wider local governance environment.

### 4.5 Cross-level linkages in local public sport facility development

The value of the framework lies in clarifying both the distinction among the three levels and their connection within the same governance process. Its logic is relational, not additive. Macro-, meso-, and micro-level concerns are not treated as parallel dimensions to be placed side by side; they are understood as interdependent elements of local public sport facility development. From this perspective, facility development is constituted through structured interdependence across policy-resource conditions, organizational mediation, and community use (14–16).

More specifically, the framework highlights three recurring forms of cross-level linkage. First, policy steering and resource conditions shape both organizational possibilities and user-level outcomes, without operating in a mechanically top-down way. Second, organizational and managerial mechanisms mediate the practical translation of broader priorities into concrete forms of provision, coordination, and use. Third, community use and user-valued outcomes generate feedback that may inform organizational adaptation while also feeding upward into public legitimacy and policy learning.

The framework should not be read as a linear sequence running from macro conditions to meso arrangements and then to micro outcomes. Its emphasis is both recursive and directional. Higher-level conditions shape lower-level possibilities, while patterns of use, perceived relevance, and experienced value may move upward through the governance process as signals of need, legitimacy, and adjustment.

This perspective clarifies why evaluation confined to one level remains insufficient. Such approaches may generate useful but partial judgments because they leave unclear how outcomes are conditioned, mediated, and fed back through the wider governance process. The value of the framework lies in showing how distinct evaluative concerns can be analytically differentiated while remaining conceptually connected within that process (14, 15).

### 4.6 Boundary conditions of the framework

The framework has clear limits of application. It is most useful for locally governed, publicly steered, and non-purely commercial sport facilities, where public goals, organizational arrangements, and community use are jointly relevant to evaluation. It is less suitable for purely private, event-led, or strongly profit-maximizing facilities whose performance is judged primarily through commercial returns or single-purpose output logics. In this respect, the framework should be treated as an analytical heuristic, not a universal managerial template.

The framework is not intended to collapse all dimensions of facility evaluation into a single composite score. Its purpose is to preserve analytical distinction while enabling integration. This matters because multidimensional public objects are often obscured when tensions among value dimensions are compressed into one metric (12, 13). The framework offers a disciplined way of working with complexity, without reducing evaluation to a simplified scoring logic.

Finally, the framework is conceptual in orientation and does not settle questions of measurement strategy, indicator selection, or causal ordering in advance. It provides an analytical basis for asking what should be distinguished, what should be connected, and why evaluation should move beyond isolated service, managerial, or efficiency indicators when local public sport facilities are examined through a governance process lens.

## 5 Discussion

### 5.1 Theoretical implications for sport facility governance research

This study contributes to sport facility governance research by giving clearer theoretical form to lines of inquiry that have often developed in parallel. Existing research has examined user experience, organizational performance, accessibility, allocation, and efficiency, although these concerns have usually remained within separate evaluative traditions (5–8, 11). The framework developed here shifts the contribution from adding another evaluative dimension to clarifying how these concerns relate within the same public development process.

One implication is that local public sport facilities should be theorized more explicitly as multi-level public service settings. That move shifts analysis away from viewing facilities only as service sites, managed entities, or investment cases in isolation. It opens a more connected line of inquiry into how user-valued outcomes, organizational mediation, and policy-resource conditions are jointly implicated in local facility development (14–16).

A second implication concerns the status of organizational mediation. Much of the literature moves too quickly from policy ambition to user outcomes, or from resource allocation to performance judgments, without giving the meso level sufficient theoretical weight. By treating organizational and managerial mechanisms as a distinct analytical level, the framework makes clearer how public priorities are interpreted, translated, and sustained in practice. This helps explain why similar policy goals or resource conditions may produce different lived outcomes across settings.

A further implication is that the framework supports a more relational mode of explanation. User experience, managerial arrangements, and public steering are not treated here as separate topics, but as mutually conditioning dimensions of the same governance process. In that sense, the framework does not replace stream-specific theories with one umbrella model. It provides an integrative architecture within which different theoretical traditions can remain distinct while being brought into clearer dialogue (12, 14).

Future theory-building in this area should move beyond the addition of isolated constructs and focus on explaining cross-level relationships. The central theoretical task is to explain how public sport facilities become usable, valued, governable, and justifiable through linked community, organizational, and policy processes.

### 5.2 Implications for local policy analysis and evaluation design

The framework has direct implications for local policy analysis and evaluation design. The central point is not that evaluation should simply incorporate more indicators, but that assessment should be organized on a clearer cross-level basis. As van der Kolk (20) argues, the effects of performance systems depend not only on whether such systems exist, but also on how they are designed and used. For local public sport facilities, this means moving beyond the expansion of already crowded performance dashboards. A more appropriate approach is to align indicators with distinct evaluative purposes and to interpret findings across levels rather than within a single domain. Facility-specific research supports this view: Iversen et al. (37) show that utilization measures may serve different governance purposes, including learning, steering, and accountability.

User-valued outcomes need to be interpreted within a broader governance context. Lived service experience, satisfaction, and perceived relevance remain important because they indicate how facilities are encountered and judged in practice. Their value is diagnostic because they show how facilities are experienced, while leaving open how those experiences are produced, sustained, or constrained across different organizational and policy settings. Their significance becomes clearer when interpreted alongside organizational arrangements and wider policy-resource conditions.

The framework also suggests that local policy analysis should approach facility development as a matter of public steering and not as a narrow provision problem. Sesinando et al. (40) show that municipalities often lack sufficiently developed instruments and data for implementation, evaluation, and monitoring. Applied to local public sport facilities, this shifts attention from whether facilities exist, how they are used, or whether they appear technically efficient, to how public priorities are translated into organizational arrangements and how policy-resource conditions shape local development. In this sense, policy analysis should focus on the conditions under which facilities become governable, justifiable, and sustainable within local systems.

Finally, the framework supports methodological pluralism without collapsing different forms of evidence into a single composite measure. Because the three levels raise distinct evaluative questions, they call for distinct forms of evidence and strategies of interpretation. User-based evidence, organizational and managerial evidence, and system-level evidence on policy steering and resource conditions should be treated as complementary, not interchangeable. This matters because simplification into one composite score may conceal as much as it reveals.

### 5.3 Implications for practice in local facility development and public steering

The framework has practical relevance for local public sport facility development, management, and public steering. For local decision-makers, the main point is that no single evaluative signal should be treated as decisive. High user satisfaction alone is not sufficient evidence that a facility is well governed, just as strong efficiency scores do not necessarily indicate accessibility, relevance, or public legitimacy. Strategic judgment requires a broader reading of how facilities perform across related evaluative domains, beyond one-dimensional scorecards.

A second practical implication is the centrality of organizational and managerial mechanisms. Public debate about sport facilities often focuses on visible provision or user outcomes, yet the viability of public goals is frequently determined at the organizational level. This is especially evident when managers respond to changing expectations about whom facilities should serve, including increasing attention to individual users in systems historically shaped by sports clubs (39). Staffing, coordination, accountability, maintenance, and operational flexibility all shape whether policy ambitions can be translated into sustained public value. In practical terms, this means that local improvement strategies must address the organizational conditions through which facilities are delivered and adapted over time, rather than focusing only on expanding provision or collecting user feedback.

A third implication concerns public steering. Public authorities need to address relevance and legitimacy alongside efficiency and utilization. Local public sport facilities are often justified through broad aims such as participation, inclusion, health, and community benefit. Those aims retain legitimacy only when they are reflected in how facilities are actually experienced and used. Accordingly, community use is more than a passive outcome measured after implementation. It provides a central reference point for governance and development decisions by showing whether broader policy intentions become meaningful in practice.

Finally, the framework makes key trade-offs more visible. De Waele et al. (13) show that performance dimensions in hybrid public organizations may reinforce one another in some situations while generating tension in others. This insight applies directly to local public sport facilities. Efforts to improve efficiency may reduce flexibility, efforts to widen access may increase organizational strain, and efforts to satisfy some user groups may generate equity concerns for others. The practical value of the framework lies in making these tensions explicit instead of allowing them to be hidden behind simplified metrics. Although this does not eliminate difficult choices, it helps decision-makers recognize them more clearly and respond more deliberately.

### 5.4 Conclusion

Evaluation of local public sport facilities remains limited when it proceeds through fragmented and single-level perspectives. Existing research has generated important insights into user experience, organizational arrangements, accessibility, allocation, and efficiency, although these strands have often developed in parallel without a shared evaluative logic. The problem is not a shortage of evaluation activity, but a lack of conceptual integration across these evaluative traditions.

This article addresses that problem by proposing a multi-level framework that approaches local public sport facilities through a governance process lens. Its contribution lies in providing an integrative architecture for understanding how user-valued outcomes, organizational mediation, and policy-resource conditions relate within the same public development process. In this view, the main shift is not the addition of more evaluative dimensions, but the reorganization of facility evaluation around a governance-process logic.

The framework is conceptual in orientation and most appropriate for locally governed, publicly steered, and non-purely commercial sport facilities. Even so, it offers a clearer basis for future research and governance-sensitive evaluation by clarifying what should be distinguished, what should be connected, and why fragmented evaluation remains inadequate for understanding local public sport facility development.
